# Christianity, Editorship, and Nurses

**Published:** 1892-02-27

**Authors:** 


### The Doctor's Armchair.

### CHRISTIANITY, EDITORSHIP, AND NURSES.

Religion and responsibility go hand-in-hand with men of sense and honesty. Christianity is so admired and esteemed in this country that many persons use it as a thing to conjure with. Not a few of these latter have little religion and less sense of responsibility. Two or three weeks ago, a newspaper with a "Christian" name published in its editorial columns the following: "The so-called Royal National Pension Fund for trained nurses seems to have practically broken down. Though backed up by the princely generosity of some city merchants, it turns out to be nothing better than a new Insurance Office, with a limited clientèle, and, consequently, with premiums that are higher than those charged by others in the same line of business."

In the language of the pulpit, we will consider the question thus raised under two heads: (1) The Editorial: (2) The Editor. "The so-called Royal National Pension Fund for trained nurses seems to have practically broken down." Why this animus against a benevolent institution which has made a free gift of £50,000 to the whole body of nurses? The Royal National Pension Fund has not stolen its title from the editor of this "Christian" newspaper, nor indeed from anybody else. It is "Royal" by the command of Her Majesty the Queen, "National," because it distributes the £2,000 a year with which it has been endowed to all the nurses in the nation who choose to become participators in its benefits, and it is a "Pension Fund," because it provides pensions for nurses at the lowest possible rates at which they can be provided, compatibly with safety and mutual benefits, to those who trust their money to its care. To dub it a "so-called" institution is to offer it a gratuitous insult, and whether an institution which gives away £2,000 a year in benevolence to trained nurses should be wantonly insulted in the editorial pages of a "so-called Christian" newspaper we must leave to the "Christian" conscience to decide.

The Pension Fund "seems to have practically broken down," says the editorial. Now what does this mean? If the fund has broken down it must have broken down in some particular way, and practically there are only two ways in which it can have broken down. Either its resources must have diminished and become inadequate to the demands that are to be made upon them, or the number of those who seek its benefits must be growing less and less. But it is the easiest thing in the world to find out both the exact amount of money which the Pension Fund has now in hand, and also the number of nurses who have applied for pensions during the last twelve months, and to compare both these sets of figures with those of the preceding twelve months. As a matter of fact the writer, who has no official connection with the Pension Fund has, for the purposes of this article, procured a copy of the annual Report of the Fund for 1890, which was issued on March 5th, 1891, and also a corrected proof of the Report for 1891, which is now being prepared, and which will be issued in the course of the next few weeks.

Now let us see what the figures for the two years say, both on the question of financial resources, and on that of the number of nurses applying for pensions during each of the contrasted years. First, we will deal with the money question. At the close of the year 1890 the total invested monies of the Pension Fund proper amounted to £73,766 0s. 1d. To this is to be added £10,000, invested for the Junius S. Morgan Benevolent Fund, making a total available for nurses' pensions and benevolent relief of £83,766 0s. 1d. in December, 1890. At the close of 1891 the amount invested on behalf of the Pension Fund proper was £96,377 10s. 5d. To this, as before, is to be added the £10,000 of the Junius S. Morgan Benevolent Fund, making a total of £106,377 10s. 5d. In other words, the Fund has so increased its resources during the past year that it is richer to day by £22,611 10s. 4d. than it was on the corresponding day of February, 1891. Is there here any indication of "breaking down" in financial resources? Can we suppose that a man who talks of breaking down in such a connection as this has the faintest glimmering of knowledge on the subject he is discussing, or the most microscopic quantity of conscience in dealing with it? And this is "Christian" editorship!

The only other circumstance which could justify the statement that the Pension Fund was "breaking down," would be distinct evidence of want of confidence on the part of nurses. But what are the actual facts on this point? During the year 1890, 524 proposals for pensions were made to the Fund, whilst 436 policies were granted. But in 1891 the number of proposals made rose to 681, and 624 policies were issued. In other words, the number of policies issued during the past year exceeded the number issued in the preceding year by 188. The actual money entrusted to the care of the Fund by nurses alone in the year that has just expired amounted to no less than £22,677 10s. 4d. Does this show any want of confidence in the Fund on the part of intelligent nurses? Does it not rather show that in spite of all the misrepresentations which folly could suggest or malignity invent, the nurses are still able to understand that it is a good thing to have a legal and indefeasible claim on the annual interest of £50,000? In short, if nurses are so foolish as to suppose that any insurance company whatever, which conducts its business on principles of openly avowed self-interest, can for a moment compete with the Royal National Pension Fund, which is conducted solely for the mutual benefit of the nurses who join it, and which besides has the annual interest of £50,000 to distribute, then nurses must be the least intelligent, not only of all women, but of all human beings whatsoever, of whom history has given us any record. That the Pension Fund has enemies we admit. But let this one fact be well and constantly borne in mind, that those enemies have everything to gain by discrediting the Fund, and nothing to lose.

So much for the "editorial" of this "Christian" newspaper, and for the avowed source of its information. The "Editor," who stands for the second head of this brief discourse, may be dismissed in a very few words. If, in charity, we admit that this gentleman is a Christian, we cannot but conclude that he is a very inexperienced and foolish one. But if we are to acquit him of inexperience and folly, then we cannot admit his Christianity. Nurses in this matter must use their own judgment. If known men of high position and trained financial capacity are not to be trusted in preference to irresponsible editors of newspapers, whose very names are not even known by their readers, then the business of the world must speedily come to an end.

